# Acetylome analysis of acetylation providing new insight into sclerotial generation in medicinal fungus *Polyporus umbellatus*

**DOI:** 10.1038/s41598-022-11798-1

**Published:** 2022-05-10

**Authors:** Bing Li, Liu Liu, Tingting Shan, Yongmei Xing, Shunxing Guo

**Affiliations:** Institute of Medicinal Plant Development, Chinese Academy of Medical Sciences and Peking Union Medical College, No. 151, Malianwa North Road, Haidian District, Beijing, 100193 China

**Keywords:** Applied microbiology, Fungal biology, Fungal systems biology, Proteomic analysis

## Abstract

Sclerotium-forming fungi are ecologically diverse and possess notable pathogenic or medicinal properties. The sclerotial generation mechanism is still elusive though *Polyporus umbellatus* sclerotia are typical Traditional Chinese Medicine with diuretic and antitumor effects. Protein acetylation displays a crucial role in several biological processes, but the functions of acetylation in this valuable fungus are unknown at present. In this study, acetylome of *P. umbellatus* was studied using nano LC-Triple TOF mass spectrometry system following immune-affinity-based enrichment. Totally, 648 acetylated sites in 342 proteins were identified and nine motifs were found to be conserved in *P. umbellatus* including K^ac^Y, K^ac^A, K^ac^L, K^ac^G, M^ac^S, M^ac^A, R^ac^A, R^ac^L, and R^ac^G. Acetylated proteins taken part in types of biological processes, particularly to those in biological processes associated with reactive oxygen species (ROS) metabolism. Inhibitors complement tests were carried out to verify the role of ROS in acetylation modification. It was concluded that oxidative stress regulated sclerotial generation via proteins acetylation in *P. umbellatus*. The present study presents new insight into the essential roles of acetylation in sclerotial formation, which may also be applicable for other sclerotium-forming fungi.

Sclerotia are a type of dense aggregations of tissue formed under adverse conditions to help fungi to survive challenging conditions^1,2^. Smith et al.^3^ reported that fungi that formed sclerotium are phylogenetically ecologically dispersed and evolutionary diverse. Sclerotium-forming fungi can be divided into two categories, the pathogenic fungi and the beneficial fungi. *Sclerotinia sclerotiorum*, a pathogen infecting over 400 plant species is ubiquitous whereas *Polyporus umbellatus* is a traditional Chinese medicine with a long history of more than 2000 years. It is widely used in diuresis, dampness permeability and anti-tumor in clinic^4^. How and why sclerotium differentiates from mycelia has always given rise to people’s interest in controlling pathogens and improving the yield of beneficial products.

The formation of sclerotium is a necessary prerequisite for medicinal material of *P. umbellatus*. Sclerotium is the form of *P. umbellatus* in the natural environment. The formation and development of sclerotium are inseparable from another fungus, *Armillaria mellea*^5^. After *P. umbellatus* sclerotia are infected by *A. mellea*, new hyphae germinate on the surface of the sclerotia, then differentiate into white sclerotia, subsequently grey sclerotia and finally develop into black and mature sclerotia, which serve as the medicinal parts. The formation and maturation of sclerotia is also the process accompanied with the accumulation of the medicinal components such as polysaccharide and ergosterol. The content of polysaccharide in black sclerotia of *P. umbellatus* can be up to 5.446%, which is significantly higher than that in gray sclerotia or white sclerotia with the contents of 3.463% and 3.315% respectively. Besides, the content of ergosterol (0.0809%) in black sclerotia is also the highest compared with that of the white and grey scelrotia^6^. For the artificial sclerotia induced in the lab, the percentage of the polysaccharide and ergosterol also rose significantly than that in the mycelia. Therefore, it is of great value to study and elaborate the secrets of sclerotial formation to yield medicinal quality components of *P. umbellatus*.

Physical conditions (such as high concentration of monosaccharide, low temperature) and symbiotic fungi *A. mellea* can affect sclerotial differentiation in combination or individually^7,8^. Regarding to sclerotial transformation directly from hyphae, many conditions such as nutrition depletion, coldness, changes of osmotic pressure or ROS affect sclerotial development. Three stages during *P. umbellatus* sclerotial formation were divided into initial sclerotia, developmental sclerotia and mature sclerotia in the previous study^9^. In filamentous fungi, sclerotial differentiation has been proved to be induced by oxidative stress. Further studies demonstrated that under the stimulation of external factors, oxidative stress has played indispensable part in sclerotial formation^10,11^. Proteomic results revealed that ROS and oxidative stress triggered the differentiation of mycelia into sclerotia in *P. umbellatus*, while the increased productions of glutathione and NAD(P)H were found to eliminate ROS for redox balancing^2^. Interestingly, proteins in these biological pathways (such as glycolysis) that led to the generation or elimination of ROS often underwent acetylated modification^12^. It was reported that protein post-translational acetylation played a key role in fungal virulence, stress response, hyphal growth, morphological transition, and metabolite production^13^. Therefore, it was not difficult to assume that oxidative stress may contribute to sclerotial generation from hyphae by regulating protein acetylation. However, it is not clear which proteins undergo acetylation modification during the differentiation of sclerotia from hyphae, and what functions they perform. This study focuses on the proteomics of the acetylated proteins in sclerotium-forming fungus.

## Results

### Generation and analysis of acetylome in *P. umbellatus*

10 data dependent acquisition (DDA) MS data were obtained in this study. Quality control results showed that the distributions of mass errors were less than 2 ppm and there was a nice correlativity between MS1 and peptide intensity or MS2 with correlation coefficient 0.98 and 0.87 fitting the accuracy requirement of MS data. The efficiency of the canonical sequence can reach to 88.2% by tryptic digestion and terminal proportion for K (Lys) and R (Arg) over 90%. A total of 342 acetylated proteins among 1382 ones (1% Global FDR from fit 0.83) were identified from hyphae and sclerotia of *P. umbellatus* (Supplementary Table S1). Of the obtained 9151 reliable peptides (1% Global FDR from fit 0.94), there were 648 acetylated ones (Supplementary Table S1), indicating that at least 7.08% of acetylated peptides lied in *P. umbellatus*.

The numbers of acetylated sites in each protein were measured to explore the distribution of these modification sites in *P. umbellatus*. The results showed that up to 63.45% of the total proteins included one acetylation site, while 36.55% were acetylated at multiple amino acids in which 18.71% and 8.48% contained two and three acetylated sites. In these acetylated proteins, 30 had four or more acetylated sites, and five contained at least 10 acetylated sites. An unknown protein (comp34876_c0) possessed 21 acetylation sites, which showed the most intense acetylation characteristic. The data above demonstrated the global research of acetylome in *P. umbellatus* for the first time.

### Functional annotation of the acetylated proteins in *P. umbellatus*

**Figure 1:**
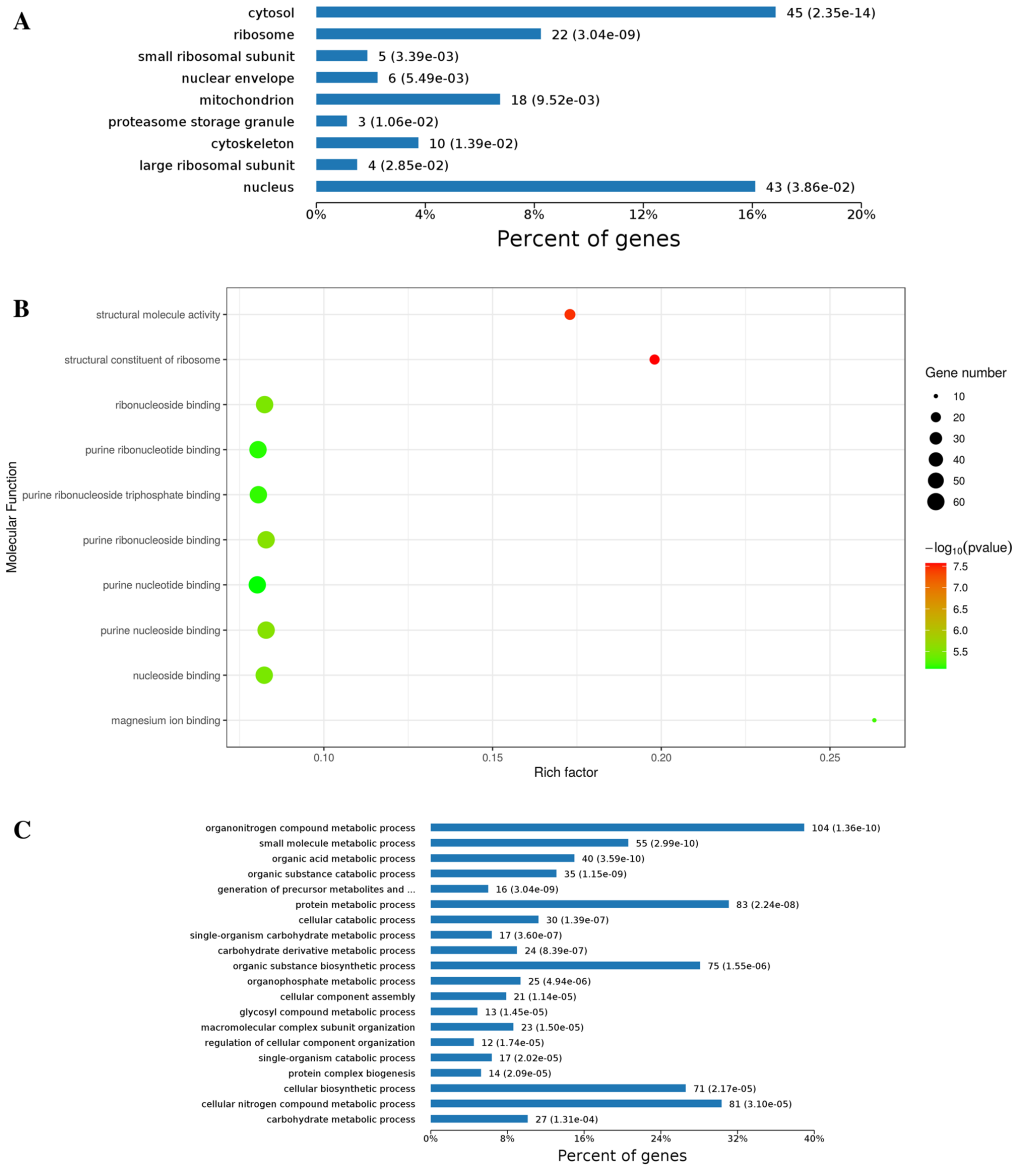
Acetylated proteins bioinformatics analysis and enrichment results in *P. umbellatus* including cellular localizations (**A**), molecular functions (**B**), and biological processes (**C**).

Gene Ontology analysis and enrichment were performed to understand the acetylome in *P. umbellatus*. A ratio of 52% acetylated proteins cannot be annotated to cell component. The others distribute in cytosol (45 proteins), ribosome (22 proteins), mitochondrion (18 proteins) and nucleus (43 proteins) predominantly. A few of acetylated in proteins located at cytoskeleton (10 proteins) and proteasome storage granule (3 proteins) (Fig. 1A).

Based on the molecular function, a great number of the acetylated proteins took part in binding functions including nucleoside binding, purine nucleotide binding and magnesium ion binding etc. (Fig. 1B). 23 and 20 acetylated proteins with higher rich factor play roles in structural constituent of ribosome, as well as structural molecule activity.

Results of biological process analysis and enrichment discovered that proteins associated with intermediate metabolism were acetylated preferentially (Fig. 1C). Those proteins were close to organonitrogen compound metabolic process (104 proteins), protein metabolic process (83 proteins), glycosyl compound metabolic process. These results indicated potential function of acetylation in regulating the process of sclerotia formation of *P. umbellatus*. This was consistent with KEGG analysis and enrichment result.

**Figure 2:**
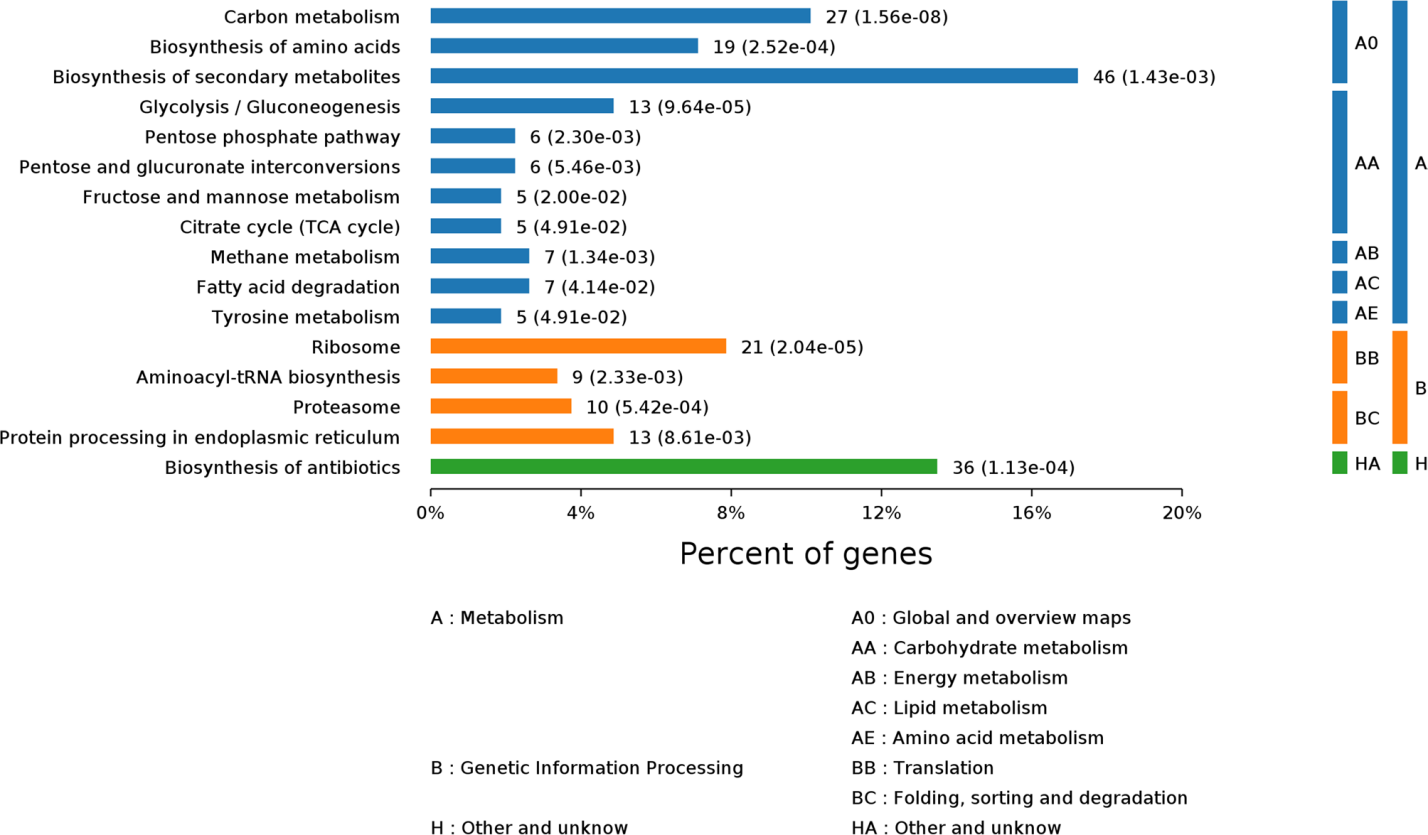
KEGG pathways of acetyl proteins in *P. umbellatus.*

Acetylated proteins in *P. umbellatus* were enriched in three subcategories by KEGG pathways enrichment analysis, including metabolism, genetic information process and other and unknown (Fig. 2). The significant pathways in metabolism subcategory included carbon metabolism (27 proteins), biosynthesis of amino acids (19 proteins), biosynthesis of secondary metabolites (46 proteins), glycolysis/gluconeogenesis (13 proteins), TCA cycle (5 proteins), and fat acid degradation (7 proteins) and so on. In addition, 21 proteins in ribosome, 10 proteins in proteasome, 9 proteins involved in aminoacyl-tRNA biosynthesis and 13 proteins participating in endoplasmic reticulum were acetylated. Besides, there were 36 acetylated proteins taking part in biosynthesis of antibiotics.

### Acetylated motifs and structural properties in *P. umbellatus*

**Figure 3:**
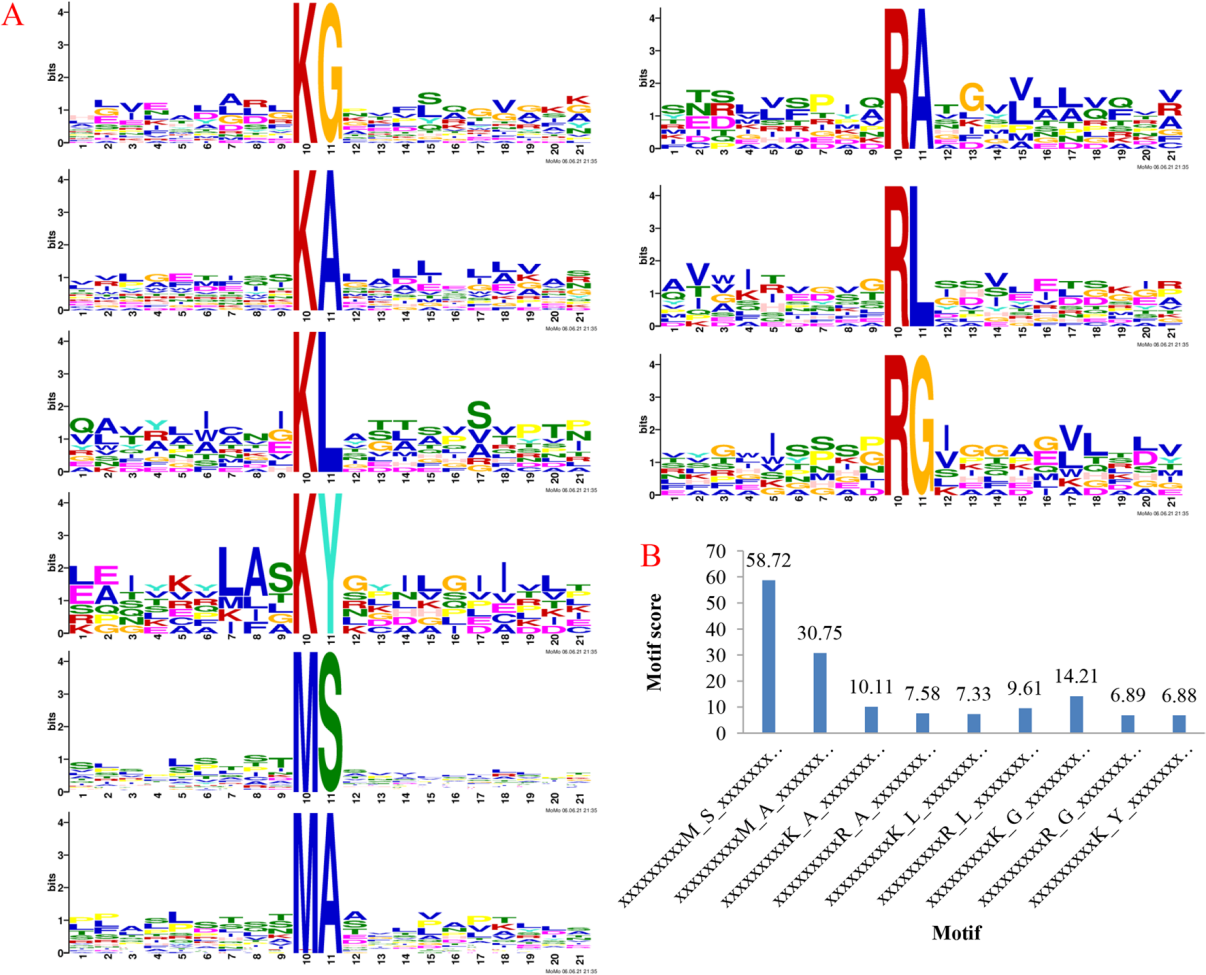
Properties of acetylation peptides in *P. umbellatus*. (**A**) Acetylated motifs and conservation of acetylated sites. (**B**) The motif scores.

Motif-X was introduced to analysis the amino acid motifs in the acetylated proteins with the identified amino residues to illustrate the characteristics of the amino acids around the identified acetylation sites in *P. umbellatus* proteins (Fig. 3, Supplementary Table S2). Of all the acetylated peptides, 147 were successfully matched to 9 conserved motifs including K^ac^Y, K^ac^A, K^ac^L, K^ac^G, M^ac^S, M^ac^A, R^ac^A, R^ac^L, and R^ac^G, where “ac” represents for the acetylated amino acid. It was obvious that the highest enrichment residues were alanine (A), leucine (L), glycine (G) and tyrosine (Y) in *P. umbellatus*. In addition, methionine (M) and arginine (R) can also be acetylated. The conserved residues were situated at the + 1 position of M and R sites and there were serine (S) and alanine (A) for Mac, followed by alanine (A), leucine (L) and glycine (G) for Rac successively.

### Acetylated protein interactions of *P. umbellatus*

**Figure 4:**
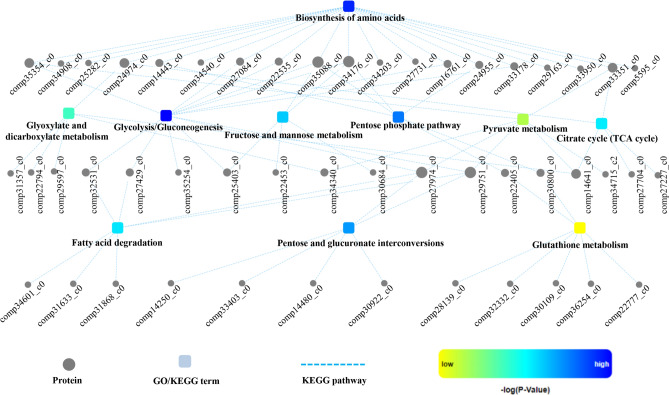
Protein–protein interaction networks of acetyl proteins of *P. umbellatus.*

To further understand the interaction of the diversity of the biological processes regulated by the acetylation in *P. umbellatus*, an acetylated protein–protein interaction (PPI) network was constructed. The PPI network showed that ten metabolic processes interacted with each other directly or indirectly (Fig. 4). For example, comp34176_c0 (6-phosphofructokinase, PFK) serves as a bridge between the biosynthesis of amino acid and glycolysis/gluconeogenesis, fructose and mannose metabolism, and pentose phosphate pathway. These pathways can be roughly divided into four categories consisting of biosynthesis of amino acids, carbohydrate metabolisms (fructose and mannose metabolism, pentose phosphate pathway, pentose and glucuronate interconversions), fatty acid degradation, and basic metabolic pathways including TCA cycle, pyruvate metabolism, glyoxylate and dicarboxylate metabolism, glycolysis/gluconeogenesis and glutathione metabolism as well. The acetylated proteins in glyoxylate and dicarboxylate metabolism (such as comp22794_c0, namely catalase, capable of eliminating H_2_O_2_) and glutathione metabolism (for example, comp30109_c0, annotated CNDP dipeptidase, involved in glutathione synthesis and recycling) were associated with redox reaction. Our previous studies proved that oxidative stress was the in-vivo inducer for sclerotia formation and development^2,11^.

### Acetylation affecting sclerotia formation of *P. umbellatus*

**Figure 5:**
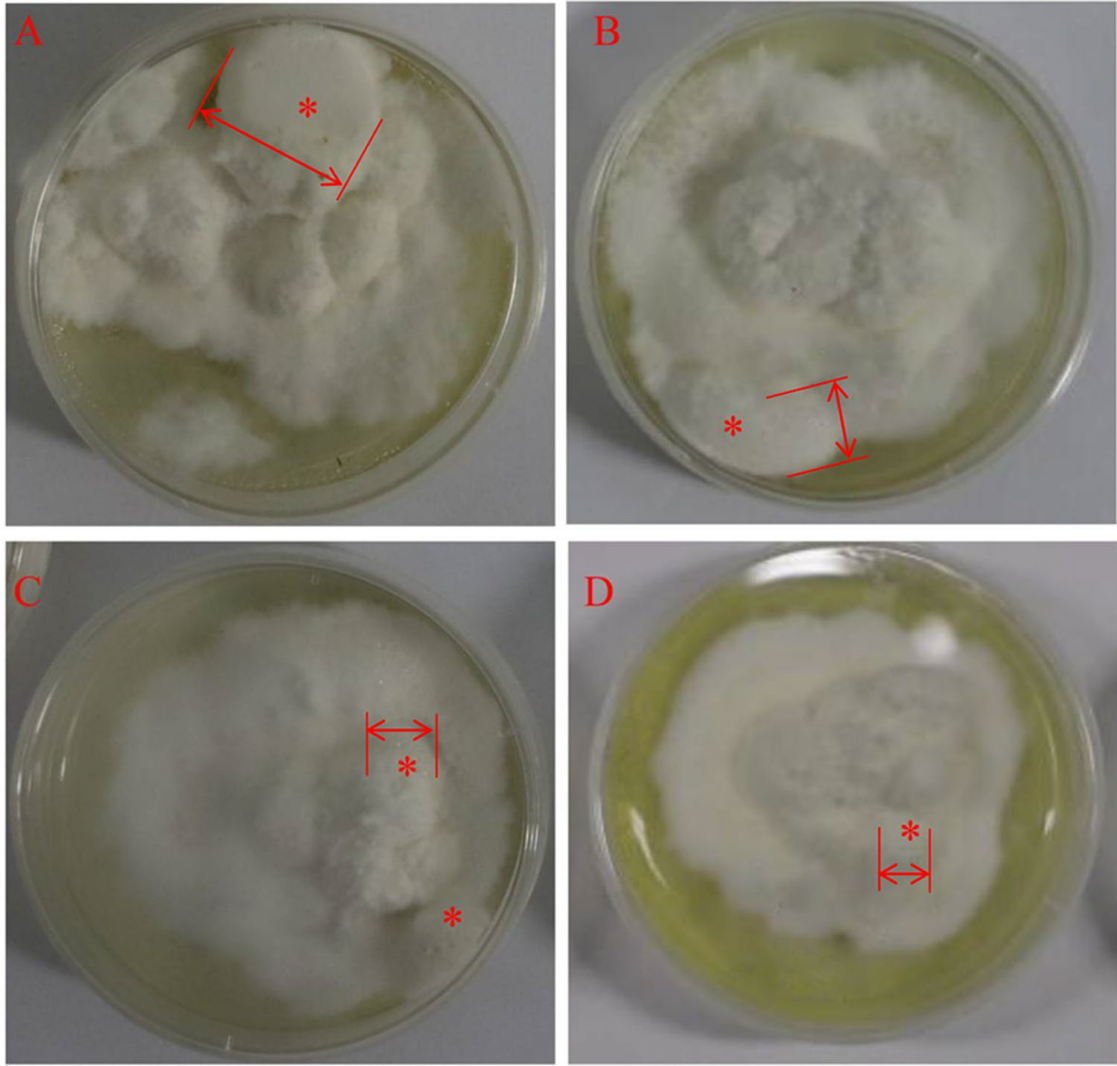
Growth characteristics of mycelia and sclerotia of *P. umbellatus* under extra treatment. (**A**) *P. umbellatus* cultured on basic medium. Sclerotia and hypha were inhibited by NADPH oxidase inhibitor DPI (**B**) and sirt1720 hydrochloride as SIRT1 inhibitor (**C**). DPI and sirt1720 hydrochloride exhibited a synergistic role in restraining *P. umbellatus* (**D**). Red asterisks represent sclerotium, and the boundary of sclerotium was marked by red double arrow.

To verify the role of protein acetylation on sclerotial formation from mycelia of *P. umbellatus*, sirt1720 hydrochloride (an inhibitor of SIRT1) were complemented into fructose complete medium to culture the fungus. *P. umbellatus* hyphae grew vigorously in the fructose complete medium (CK). Sclerotia can be differentiated from hyphae after inoculation for 30 days with 61.1% rate of sclerotia-formation, and the average diameters and fresh weight of initial sclerotia were 16.0 ± 3.74 mm and 0.262 ± 0.147 g (n = 5) respectively (Fig. 5A, Table 1). On contrast, sirt1720 hydrochloride could inhibit the hyphae growth of *P. umbellatus* in comparison with the CK. Furthermore, the formation time of sclerotia was delayed with an average of 13 days later than that of the CK, with the sclerotia-formation rate (40.0%) were also less than CK resulting in sclerotia diameter of 7.79 ± 1.38 mm and fresh weight 0.131 ± 0699 g (n = 5) (Fig. 5B, Table 1). These results showed that protein post-transcriptional acetylation contributed to sclerotial generation from hyphae of *P. umbellatus*.

**Table 1:** Statistic results of sclerotia after added inhibitors.

	Rate of sclerotial formation (%)	Diameters of sclerotia (mm)	Fresh weight of sclerotia (g)
CK	61.1	16.0 ± 3.74	0.262 ± 0.147
DPI group	47.2	7.78 ± 2.37**	0.161 ± 0.050*
SIRT1(–) group	40.0	7.79 ± 1.38**	0.131 ± 0699*
DPI and SIRT1(–) group	33.3	10.5 ± 0.880**	0.181 ± 0348*

SIRT (–) mean added SIRT inhibitor sirt1720 hydrochloride. * and ** represented there were difference and significant difference between the group with CK.

After being treated with 250 μM DPI, the inhibitor of NADPH oxidase, the mycelial growth rate was lower than that of the CK, and the aerial hyphae in the DPI complement group were not as vigorous as those in the CK. Obviously, the sclerotium was inhibited by DPI, as the sclerotial formation time was postponed to 38th days with the sclerotial formation rate 47.2%. The average sclerotial diameters and fresh weight were 7.78 ± 2.37 mm (n = 5) and 0.161 ± 0.050 g (n = 5) respectively which were significantly less than that of the CK (Table 1, Fig. 5C).

By comparison with the CK, combined with 250 μM DPI and 11.9 μM sirt1720 (SIRT1 inhibitor), the sclerotium-forming rates of *P. umbellatus* were only 33.0%, the diameters of sclerotia were 10.5 ± 0.880 mm, and the fresh weight were 0.181 ± 0348 g, indicating that the sclerotia formation and growth of *P. umbellatus* were both inhibited. The inhibitor complement studies also showed that acetylation and ROS could regulate the formation of sclerotia simultaneously (Fig. 5D, Table 1).

**Figure 6:**
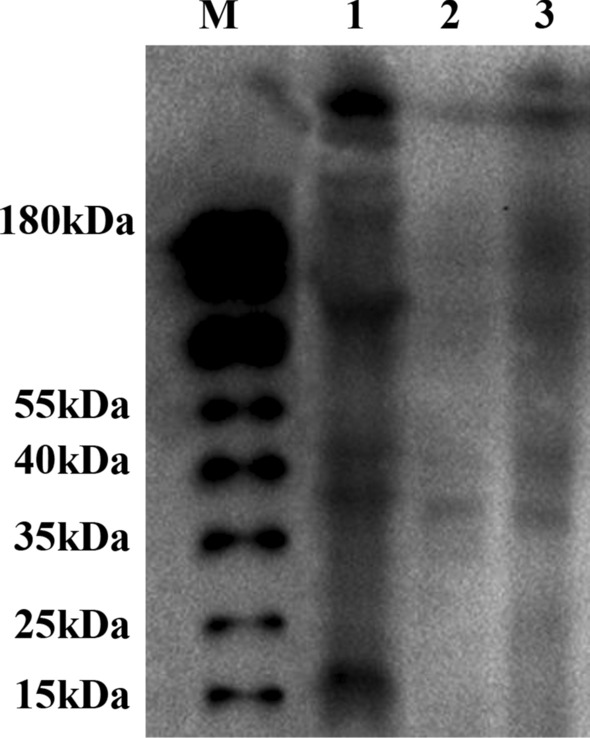
Western blotting results of mycelia and sclerotia for control group and DPI added group. M: marker, 1 and 2 were mycelia and sclerotia in control group respectively, and 3 represents mycelia in DPI added proteins. The raw digital image was uploaded as Supplementary Figure.

The post-translational acetylation proteins were qualitatively detected by western blotting (WB). WB results (Fig. 6) intuitively presented the difference between DPI added group and the CK. There were 8 obvious bright bands, the acetylation modified proteins in mycelia, while the gray scale of acetylated proteins in sclerotia were less than that in mycelia indicating that protein acetylation is conducive to sclerotial formation in *P. umbellatus*. After DPI added, the numbers of bands in mycelia were less than those in mycelia of the CK, and the gray scale of acetylation proteins was significantly weaker too, which showed that the acetyl modification was weakened.

Our previous studies demonstrated that NADPH oxidase is the key enzyme for the production of ROS, and DPI (the NADPH oxidase inhibitor) inhibited the formation of sclerotia^2,11^. Therefore, ROS might regulate the formation of sclerotia by mediating protein acetylation modification *P. umbellatus*.

### Expression levels of acetylated modification proteins by PRM

The expression of the acetyl proteins related to the generation or elimination of ROS in the protein–protein interaction network were quantified by PRM. One specific peptide for each targeted protein was selected to measure the peak areas and 23 acetylated proteins were quantified successfully in this study (Supplementary Table S3). Peak areas were normalized, and then the relative changes of acetylated proteins were calculated between mycelia and sclerotia in CK or mycelia in DPI-complemented group (Table 2). Mycelia were the tissue for accumulation of ROS which induced sclerotia. Adding DPI can regulate the expression levels of acetyl targeted proteins in mycelia (IH_DPI_/IH_CK_) (Table 2), in which most proteins were up-regulated, such as glucose-6-P dehydrogenase (G6PD) (IH_DPI_/IH_CK_ 1.62) and 6-phosphogluconate dehydrogenase (6-PGD) (IH_DPI_/IH_CK_ 2.80) in glutathione metabolism and glycate and dicarboxylate pathway. It was not conducive to the accumulation of ROS in mycelia, which affected the formation of sclerotia of *P. umbellatus*.

**Table 2:** Expression levels of targeted acetylation proteins in *P. umbellatus.*

Acetylated protein	Abbreviated name	ID	Ratio by PRM	
			IS/IH	IH_DPI_/IH_CK_
Hexokinase-B	Hexokinase-B	comp25403_c0	1.28	1.69
6-phosphofructokinase	PFK	comp34176_c0	2.00	1.71
Fructose-bisphosphate aldolase	FBA	comp35088_c0	1.75	1.06
Glyceraldehyde-3-phosphate dehydrogenase	GAPDH	comp33178_c0	2.11	1.87
Phosphoglycerate kinase	PGK	comp16761_c0	3.24	2.29
Cofactor-independent phosphoglycerate mutase	GPMI	comp22535_c0	2.01	1.46
Pyruvate decarboxylase	PDCA	comp35254_c0	1.32	1.47
Aldehyde dehydrogenase	ALDDH	comp27974_c0	1.63	2.21
GroES-like protein	ADH	comp32531_c0	1.52	1.33
Glucose-6-P dehydrogenase	G6PD	comp30800_c1	1.15	1.62
6-phosphogluconate dehydrogenase	6-PGD	comp22405_c0	1.46	2.80
6-phosphofructokinase	ATP-PFK	comp34176_c0	2.00	1.71
Pyruvate carboxylase	PCB	comp33351_c0	2.25	0.47
ATP-citrate synthase	ACL	comp27227_c0	2.9	1.49
Succinate-CoA ligase	SCS-beta	comp27704_c0	2.03	1.76
Malate dehydrogenase	MDH	comp14641_c0	1.62	1.09
Malate synthase	Malate synthase	comp34340_c0	1.05	8.31
Acetyl CoA carboxylase	ACC	comp34715_c2	1.44	1.86
NAD-aldehyde dehydrogenase	ALDHIII	comp29751_c0	1.43	0.76
5-oxoprolinase	5-oxoprolinase	comp28139_c0	1.29	1.58
CNDP dipeptidase	DUG1	comp30109_c0	1.50	1.44
Glutathione S-transferase C-terminal-like protein	GST N1-1	comp22777_c0	1.27	0.96
Enoyl-CoA hydratase	SCEH	comp31086_c0	1.19	1.32

## Discussion

Sclerotium bestows fungi increased resistance to adverse environment. Some medicinal fungal sclerotia are valuable for human being, such as *P*. *umbellatus*, while pathogenic sclerotia can cause severe economic loss for example *S. sclerotiorum*. Sclerotium differentiated from hyphae, but the generation mechanism is elusive. Our previously studies demonstrated that oxidative stress plays crucial roles in triggering sclerotial formation from mycelia, while antioxidant activity is vital for cell survival from oxidative stress^2,10,11^, and some differently expressed proteins involved in this process, such as pyruvate dehydrogenase (PDH) in glycolysis/gluconeogenesis, isocitrate dehydrogenase subunit (IDH) in TCA cycle^2^. Zhao et al.^12^ demonstrated that those proteins can be acetylated and regulated cellular metabolism in response to extracellular conditions. Therefore, it is necessary to systematically study the acetylome and its contribution to sclerotium generation of *P. umbellatus*.

Acetylation is one of post-translational modification of proteins, which widely exists in animals, plants and microorganisms. Protein acetylation modification has attracted extensive attention and research because it is involved in many important physiological functions, such as transcription regulation, signal transduction, cell division, stress response and even pathogenic microbial infection regulation^14^. In this study, we identified four types of lysine acetylated motifs, including K^ac^Y, K^ac^A, K^ac^L and K^ac^G. Acetylated lysine (Kac) motifs K^ac^Y was observed in eukaryotes such as in human cells and grape, representing regulatory pathways of energy metabolism (TCA cycle, oxidative phosphorylation, lipid metabolism) in mammals and plant defense respectively^15,16^. This motif also exists in prokaryotes, like *E. coli*, *Vibrio parahemolyticus*, *Brenneria nigrifluens*^17–19^ and functioned in stress response. K^ac^L was also uncovered in *Brenneria nigrifluens*. K^ac^A and K^ac^G are rarely identified in other microbe species. Acetylation of methionine (Mac) and arginine (Rac) was another kind of important modification in the formation process of sclerotia in *P. umbellatus*, which has been rarely reported in other species. Srinivasan et al. reported that methionine acetylation (Mac) can regulate the stability of phosducin like 3 (PDCL-3)^20^. High expression levels of PDCL-3 increased the sensitivity to hypoxia stress and impacted on the interaction with vascular endogenous growth factor receptor 2 (VEGFR-2) leading to regulating angiogenesis in Zebrafish and mouse. In *E. coli*, acetylation of arginine site of ribosomal protein L12 not only affected the regulation of cell cycle, but also enhanced the stress resistance of bacteria by enhancing the intra-molecular interaction of ribosomal stalk^21^. Sclerotia formation is to adapt to the environment change. At present, the specific functions of these two kinds of motifs (Mac and Rac) in this process are still unclear, but it can be inferred that they can enhance the sensitivity and resistance of *P. umbellatus* to adverse stress.

Oxidative stress may regulate the formation of *P. umbellatus* sclerotia through protein post-translational modified acetylation. On the one hand, deacetylase inhibitors (sirt1720 hydrochloride) inhibited the formation of *P. umbellatus* sclerotia. Deacetylase can hydrolyze protein acetyl groups, and activating deacetylase can increase the adaptability to adverse environment^19^. In this study, when sirt1720 hydrochloride, a deacetylase inhibitor of SIRT1, was added, the rate of sclerotial formation, the time of sclerotial appearance and fresh weight of *P. umbellatus* sclerotia were even lower than those in the CK (Table 1 and Fig. 5). These results showed that protein post-translational acetylation can regulate *P. umbellatus* sclerotial generation. On the other hand, when *P. umbellatus* encounters adverse stress (high concentration maltose such as 50 g/L)^7,10^, ROS accumulates in the hyphae^11^ and promotes the formation of sclerotia by activating MAPK signaling pathway^22^. After NADPH oxidase inhibitor DPI was added, the sclerotial formation rates were lower than that of the CK. Therefore, ROS might regulate the formation of sclerotia by mediating protein acetylation modification *P. umbellatus*.

**Figure 7:**
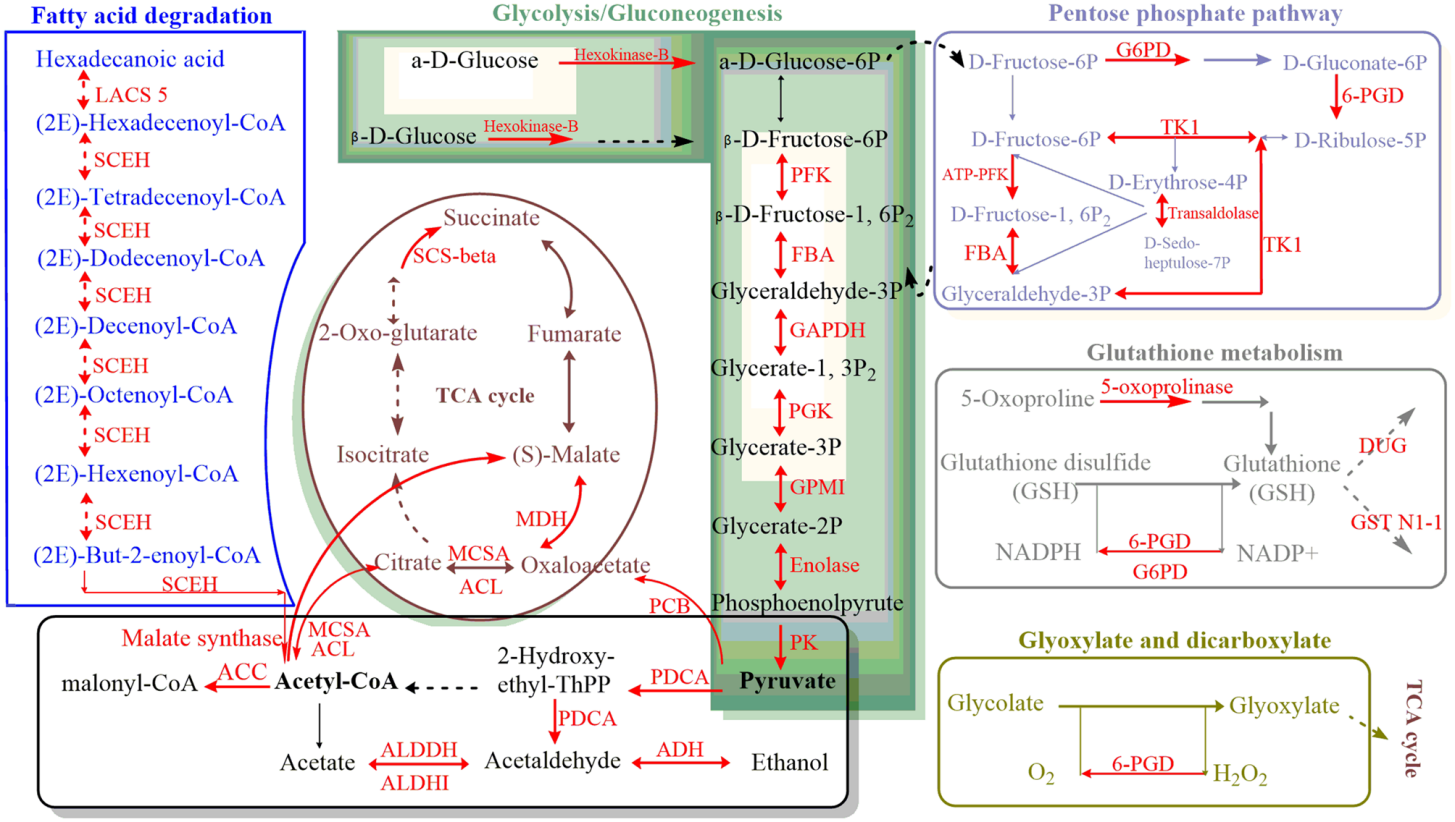
Quantified acetylation proteins by PRM related to the generation or elimination of reactive oxygen species in the protein–protein interaction network in *P. umbellatus*. Red arrows and words represent acetylated proteins.

Western blotting results also showed that there were significant differences in the acetylated proteins of the mycelia and sclerotia in the group and DPI-supplemented group, that was, ROS would affect the protein post-translational acetylation as well. Meanwhile, the generation and scavenging of the ROS levels are maintained balanced in the process of *P. umbellatus* sclerotial differentiation^2^, in which glutathione (GSH) is an important antioxidant substance^23^. When sclerotia generated from mycelia of *P. umbellatus*, some key enzymes in glutathione metabolism, glyoxylate and dicarboxylate pathway were acetylated, such as 5-oxoprolinase in the process of GSH synthesis, G6PD regulating NADPH production and 6-PGD, they all play important roles in reducing hydrogen peroxide (Fig. 7). Their common feature was to regulate the accumulation of antioxidant GSH and the clearance of oxidative substances. These two biological processes are also directly or indirectly associated with TCA cycle, glycolysis/gluconeogenesis, fat acid generation, pyruvate metabolism and pentose phosphate pathways (Figs. 4, 7). Proteins in these cellular biological processes were not only acetylated, but also up-regulated in sclerotia of the CK or in mycelia of DPI added group (Table 2), which indicated that ROS could affect the sclerotial formation of *P. umbellatus* by regulating the acetylation or the expression levels of these acetylated protein.

## Methods

### *Polyporus umbellatus* hyphae and sclerotia culture

Fructose complete medium was used to culture *P. umbellatus* in dark as previously described^2,7^. *P. umbellatus*’ hyphae and sclerotia were collected at the 30th day after inoculated and defined as IH and IS respectively. Three biological replicates were harvested from each group.

In order to verify oxidative stress and acetylation contribution to sclerotia formation, NADPH oxidase and deacetylase Sirtuin1 (SIRT1) inhibitor complement tests were conducted. 250 μmol/L diphenyleneiodonium chloride (DPI) as NADPH oxidase inhibitor or sirt1720 hydrochloride as SIRT1 inhibitor were added to the fructose complete medium to culture *P. umbellatus*, which were defined as the DPI group and SIRT1(–) group respectively. The group with DPI and sirt1720 hydrochloride complement simultaneously referred to as the DPI-SIRT1(–) group, while the CK was without any inhibitors addition. The time of sclerotial appearance was recorded, and the diameters of the mycelia and fresh weight of sclerotia were also measured. Importantly, sclerotial formation rates were calculated with the ratio numbers of petri dishes that formed sclerotia to the total numbers of the inoculated petri dishes.

### Peptides preparation and immune-precipitation

Peptides of hyphae and sclerotia were prepared as our previous study^2^, and the main procedures were as follow: after frozen in liquid nitrogen, fresh hyphae or sclerotia were ground into powder, and protein extraction solutions for fungi were added to extract the total proteins. Subsequently, the crude proteins were purified with 20% trichloroacetic acid-acetone solution, and then the purified proteins were reduced with 10 mM Tris (2-carboxyethyl) phosphine (BOC Sciences) at 60 °C for 30 min and alkylated with 10 mM iodoacetamide (Sigma) in the dark for 30 min at room temperature successively following generating protein tryptic fragments by adding modified trypsin (Promega) at 1: 40 protein-to enzyme at 37 °C.

The trypsinized peptides were separated into fractions by HPLC using Gemini-NX C18 column (4.6 mm × 250 mm, 5 μm 110 Å; phenomenex, USA). Briefly, the fragments were loaded on column, and eluted with a gradient of 5% to 60% acetonitrile in 20 mM NH_4_CO_3_ (pH 10) for 30 min, and then 60% to 90% for 5 min resulting in 20 collected elution fractions. Then, fractions were combined into 10 samples and dried by lyophilization.

Anti-acetyllysine antibody conjugated agarose beads (Jingjie PTM Bio, China) were used to enrich the acetylated peptides. At first, re-dissolved the dried fractions with NETN buffer (100 mM NaCl, 1 mM EDTA, 50 mM Tris–HCl, and 0.5% NP-40, pH 8.0). Then, adding beads for incubating overnight with gentle end-to-end rotation at 4 °C. After that, antibody conjugated beads was washed with 0.5 ml NETN buffer, ETN buffer and Milli-Q water sequentially by inverting tube for 15 times, and repeated three times. The combined beads were eluted with 0.1% trifluoroacetic acid, and then subjected to vacuum-dried to obtain acetyl peptides finally.

### LC–MS/MS data acquisition and acetylome identification

Nano LC-Triple TOF 5600 + system was used to acquire LC–MS/MS data. The major protocol was referred to our previous study^2^. In general, acetylated peptides were injected into nano LC system to desalt on loading trap column (350 μm × 0.5 mm, Chrom XP C18-3 μm, 120 Å, Eksigent) and then separated by nano analytical column (75 μm × 20 cm, Sunchrom C18-5 μm, 120 Å, Beijing Happy Science Scientific Co. Ltd). After that, the sample elution was sprayed, ionized and the MS signal were acquired by Triple TOF 5600 + platform on data dependent acquisition (DDA) model. Excepting for the MS1 spectra ranging from *m/z* 350 to 1250, the rest parameters for the spectra data acquisition were the same with our previous study^2^.

The DDA data were evaluated and analyzed by ProteinPilot™ (AB Sciex) against theoretical protein library (.fasta file) translated from *P*. *umbellatus* transcriptome to identify acetylated proteins^24^. All the DDA data generated an acetylome of *P*. *umbellatus* and the parameter “special factors” in ProteinPilot was set “acetylation” the same with others in our previous report^2^.

### Acetylomic Motif and bioinformatic analysis

Potential motifs of *P*. *umbellatus* acetylome were analyzed and generated using Momo Motif-X model online tool^25^. Algorithm settings were listed as follow: the motif width was set 21 with 10 upstream and downstream amino acids in a special site; the minimum occurrences for residue/position pair 1; the binomial probability threshold for residue/position pair 0.000001; the binomial p-value calculations was selected “accurate”; the theoretical protein library for acetylome identification were used as background-database.

As there was no genome database, *P*. *umbellatus* acetylome was blasted with homologous species *Phanerochaete carnosa*. Gene Ontology (GO)^26^ annotation was analyzed and enriched by Omicsbean™ online tool, so as Kyoto Encyclopedia of Genes and Genomes (KEGG)^27^ and protein–protein interactions.

### Expression levels of acetylated proteins measure by PRM

To further verify the expression levels of acetylated proteins taking part in ROS and acetylation, candidate proteins were measured using parallel reaction monitoring (PRM) method following the previous procedures^28^. The main steps were as follow: The peptides of hyphae and sclerotia were prepared with the final concentrations of 1 μg/μL. Peptides were separated by two dimensional chromatographic systems, and then the PRM data were acquired on UltiMate3000 RSLC nano LC (Dionex, CA) and Orbitrap Fusion (Thermo-Fisher Scientific, CA) mass spectrometry system. Parameters or settings for PRM acquisition and processing were consistent with the previous study^28^.

### Western blot analysis

The previously extracted proteins were mixed with protein loading buffer, boiled, and centrifuged at 10 000 × g for 60 s. Then, protein samples solution with total 60 μg proteins samples were separated by SDS-PAGE on a 10% gel and transferred to nitrocellulose membrane (Solarbio Life Science), then blocked with 5% skim milk at 25 °C for 1 h. Pan acetyllysine antibody (Jingjie PTM BioLab Co. Ltd) (1:1000) was incubated overnight at 4 °C. Subsequently, the membrane was washed in triplicate with 1 × TBST, 10 min each following incubated with Goat Anti-Mouse IgG H&L (HRP) (Solarbio Life Science) (1:5000) at 25 °C for 1 h. Subsequently, the membrane was washed as described above. Finally, antibody binding was detected by chemiluminescence.

## Conclusion

In the present study, acetylated sites and proteins were identified using nano LC-Triple TOF mass spectrometry system following immune-affinity-based enrichment. Totally, 648 acetyl sites in 342 proteins were identified from the hyphae and sclerotia of *P. umbellatus*. *P*. *umbellatus* possesses types of conserved motifs including K^ac^X, M^ac^X and R^ac^X (X represents high frequency amino acids). These modified proteins were related to types of biological processes, especially to those proteins associated with ROS metabolism. Further tests results demonstrated that ROS regulated sclerotia generation from hyphae via proteins acetylation in *P*. *umbellatus*. These results encourage further investigation of mechanisms and methods to control the growth of pathogenic fungus or to promote the formation of sclerotia of valuable fungus.

## Supplementary Information

Supplementary Figure 1.

Supplementary Tables.

## Data Availability

The acetylome and PRM datasets generated during the current study are available in the ProteomeXchange Consortium (http://proteomecentral.proteomexchange.org) via the PRIDE partner repository with the dataset identifier PXD031195 (username: reviewer_pxd031195@ebi.ac.uk, password: a3tzAtm5) and PXD031198 (username: reviewer_pxd031198@ebi.ac.uk, password: 97Seu503).
