# Supplementary material for: Acetylome analysis of acetylation providing new insight into sclerotial generation in medicinal fungus Polyporus umbellatus
Source: Sci Rep. 2022 May 10;12:7629. doi: 10.1038/s41598-022-11798-1 (PMC9090770; doi:10.1038/s41598-022-11798-1)
Supplement: Supplementary file 1 — Supplementary Figure 1. [file 41598_2022_11798_MOESM1_ESM.docx]

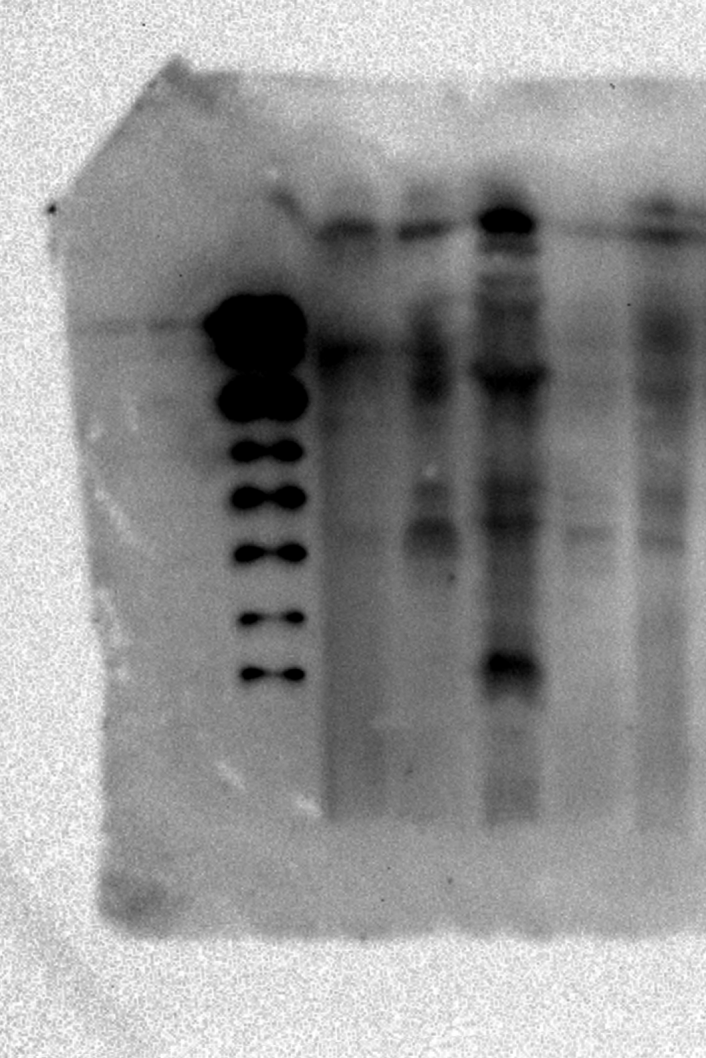


Supplementary Info File 2. The raw digital image of western blotting. From the left to right, each bond was loaded 10 μg protein markers, 10 μg proteins of mycelia in CK group, 20 μg proteins of mycelia in CK group, 60 μg proteins of mycelia in CK group, 60 μg proteins of sclerotia in CK group and 60 μg proteins of mycelia in DPI-added group. The exposure time was 49.3s.
